# Pediatric COVID-19: clinical and epidemiological data of 1303 cases in a general hospital in Brazil

**DOI:** 10.1590/1984-0462/2024/42/2023031

**Published:** 2023-12-22

**Authors:** Vera Bain, Marcelo Luiz Abramczyk, Ricardo Luiz Soares Costa, Milena Ribeiro Paixão, José Leão de Souza

**Affiliations:** a Hospital Israelita Albert Einstein, São Paulo, SP, Brazil.

**Keywords:** COVID-19, SARS-CoV-2, Child, Adolescents, COVID-19, SARS-CoV-2, Crianças, Adolescentes

## Abstract

**Objective:**

This study aimed to describe the clinical characteristics of the pediatric population with COVID-19 in an Emergency Department (ED) of a Brazilian general hospital.

**Methods:**

Epidemiological and clinical data of patients younger than 20 years old were collected from patients’ medical records from February 2020 to July 2021. Most of the epidemiological data described pertains to hospitalized patients. We also reviewed coinfections, treatment, and outcomes and compared the first and second waves of COVID-19.

**Results:**

We identified a total of 1303 episodes of SARS-CoV-2 infection. The median time from symptom onset to diagnosis was three days. Symptoms were present in 92.3% of the patients. The most common symptoms were fever (45.2%), nasal congestion/discharge (44.2%), and cough (39.4%). Chest radiography and tomography were performed in 7.7 and 3.3% of cases, with abnormal findings in 29.7 and 53.4%, respectively. Hospital admissions occurred in 3.5% of patients, mainly in the presence of comorbidities, in children under five years old and in those who presented to the ED during the first wave of COVID-19. Coinfection with a viral agent was identified in 20% of the 71 cases tested in this study, and a positive rapid test for *Streptococcus pyogenes* was found in 8% of the 174 cases tested, with no impact of these coinfections on hospitalization.

**Conclusions:**

We found that COVID-19 was a mild disease in most children in our study population, with most hospitalizations and readmissions occurring during the first wave of COVID-19.

## RESUMO

**Objetivo:**

Este estudo teve como objetivo descrever as características clínicas da população pediátrica com COVID-19 no setor de emergência de um hospital geral brasileiro.

**Métodos:**

Os dados epidemiológicos e clínicos de pacientes idade inferior a 20 anos foram coletados dos prontuários dos pacientes de fevereiro de 2020 a julho de 2021. A maior parte dos dados epidemiológicos descritos refere-se a pacientes hospitalizados. Também foram revisados as coinfecções, o tratamento e os desfechos de internação e comparadas a primeira e a segunda ondas de COVID-19.

**Resultados:**

Foi identificado o total de 1.303 episódios de infecção por SARS-CoV-2. A mediana de tempo desde o início dos sintomas até o diagnóstico foi de três dias. Os sintomas estiveram presentes em 92,3% dos pacientes. Os sintomas mais comuns foram febre (45,2%), congestão/secreção nasal (44,2%) e tosse (39,4%). Radiografia e tomografia de tórax foram realizadas em 7,7 e 3,3% dos casos, com achados anormais em 29,7 e 53,4%, respectivamente. As internações hospitalares ocorreram em 3,5% dos pacientes, principalmente na presença de comorbidades, em crianças menores de cinco anos e naqueles que se apresentaram no pronto-socorro durante a primeira onda de COVID-19. Foi identificada coinfecção viral em 20% dos 71 casos testados e teste rápido positivo para *Streptococcus pyogenes* em 8% dos 174 casos testados, não havendo impacto dessas coinfecções quanto à internação hospitalar.

**Conclusões:**

Observamos que a COVID-19 foi uma doença leve na maioria das crianças de nossa população, com a maior parte das hospitalizações e readmissões ocorrendo durante a primeira onda de COVID-19.

## INTRODUCTION

The coronavirus disease 2019 (COVID-19) is a multisystemic disease that can lead to respiratory failure, exacerbated inflammatory response, and death.^1^ Children and adolescents usually have a mild disease similar to other respiratory viral infections.^2^ Nevertheless, a small percentage of pediatric patients may experience severe pulmonary disease or develop multisystemic inflammatory syndrome in children (MIS-C).^2,3^ MIS-C is a potentially life-threatening condition that can manifest as organ failure, Kawasaki-like symptoms, and shock.^3^ Several risk factors have been identified in the pediatric population, including obesity, diabetes, asthma, genetic disorders, neurologic disorders, and younger age (mainly newborns).^4,5^

In Brazil, the impact of the pandemic has been significant, with high rates of infection, hospitalization, and deaths.^5–7^ Most studies in Brazilian children and adolescents are based on public access information about hospitalizations, and few studies provided detailed clinical information based on medical records.^5,7–11^ Therefore, the purpose of the present study was to describe the clinical features of children and adolescents with COVID-19 who sought care at the emergency department (ED) of a Brazilian general hospital.

## METHOD

This single-center retrospective study was conducted at the ED of Hospital Israelita Albert Einstein, a private general hospital in Sao Paulo, Brazil, catering to a high socioeconomic status population. The Institutional Review Board approved the study (protocol n. 51476921.3.0000.0071) and a waiver of consent. The data were obtained in the institutional digital medical records. All the procedures were performed following the ethical standards of the Albert Einstein Hospital research committee and the 1964 Helsinki declaration and its later amendments.

All patients below 20 years of age with a positive reverse transcription polymerase chain reaction (RT-PCR) or antigen test for SARS-CoV-2 who presented to the ED between February 2020 and July 2021 were included in the study. No exclusion criteria were applied in this study. We reviewed the demographic and clinical characteristics, such as sex (masculine/feminine), age (calculated based on admission date and birthdate), signs and symptoms (such as fever, myalgia, arthralgia, headache, skin lesions, malaise/asthenia, retro-orbital pain, conjunctivitis, coryza, nasal congestion, ear pain, painful swallowing, cough, hyposmia/anosmia, hypogeusia/ageusia, shortness of breath, wheezing, chest pain, nausea, vomiting, loss of appetite, diarrhea, abdominal pain), chronic medical conditions referred during the ED visit (such as asthma, diabetes, obesity, neurologic disease, heart disease, genetic condition, immunosuppression), history of contact with a confirmed or suspected case of COVID-19 reported by patient or caregiver, and results of complementary laboratory and imaging tests.

While weight was assessed for all ED patients, height was not documented in our records, precluding the calculation of body mass index. Emergency physicians were recommended to inquire about history of a contact with suspected or confirmed COVID-19 cases for all patients throughout the pandemic. Physical examination was performed by the ED physicians according to their regular practice, while laboratory exams and radiological images were performed according to clinical judgment and updated guidelines. The reference values used to interpret the laboratory results were based on the normal ranges established by hospital laboratory service, which are provided alongside the corresponding results in each table. In our hospital ED, chest radiographs were initially analyzed by the attending physician and reviewed with a radiologist in case of any interpretation uncertainties. Other radiological exams are directly evaluated by a radiologist. Additionally, we assessed the number of visits to the ED and hospitalization rates.

To investigate the association between clinical and laboratorial data of pediatric patients with COVID-19 and rates of hospitalization and readmission to the ED, patients were categorized into three age groups: 0–5 years old, 6–11 years old, and 12–19 years old. Furthermore, we sought to assess if there were differences in clinical presentation and severity of COVID-19 infection during the early stages of the pandemic. For this purpose, we examined Brazil’s first wave of COVID-19, taking place for the whole of 2020, and the second wave that began in January 2021. We compared the clinical characteristics and outcomes of children and adolescents who presented to our ED during these distinct waves.

The data were described using absolute and relative frequencies for categorical variables, and means, standard deviations, medians, quartiles, minimum and maximum values for numerical variables. The distributions of numerical variables were assessed using histograms and boxplots, as well as Shapiro-Wilk tests for normality. The relationships between categorical variables were investigated using Chi-square tests or Fisher’s exact tests, depending on the observed sample distribution. To compare the study periods in relation to patient age, the non-parametric Mann-Whitney test was utilized. The data analysis was performed using Statistical Package for the Social Sciences (SPSS) v24.0.

## RESULTS

We identified 1303 episodes of SARS-CoV-2 infection during the chosen period among 1299 patients (aged zero to 19 years). The demographic data are described in Table 1.

**Table 1:** Epidemiological characteristics of children and adolescents with SARS-CoV-2 infection in a Brazilian private hospital (n=1303).

Characteristics		n	%
Age, years (median, IQR)		13.0 (7.0–17.0)	
	0–5	273	21.0
	6–11	275	21.1
	≥12	755	57.9
Male sex		642	49.3
Indication for testing*			
	Signs and symptoms of infection	1203	92.3
	Contact with a case of COVID-19	815	62.5
	Possible contact with COVID-19	117	9.0
	Other	13	1.0
Time from symptom onset to test, days		3.0±2.5	
	0–3 days	836	64.2
	≥4 days	361	27.7
Comorbidities		122	9.4
	Asthma	81	6.2
	Diabetes	2	0.2
	Obesity	6	0.5
	Neurologic disorders†	14	1.1
	Cardiac diseases‡	12	0.9
	Genetic syndrome§	7	0.5
	Immunosuppression//	6	0.5

Results are presented in n (%).

* indication for testing was defined by the attending physician;

† Neurologic disorders: epilepsy n=9; cerebral palsy n=2; autism n=2; *Miastenia gravis* n=1;

‡ Cardiac diseases: congenital heart disease n=11; coronary aneurism post Kawasaki disease n=1;

§ Genetic syndrome: Down syndrome n=3; other rare syndromes n=4;

// Immunosuppression: Chron disease n=3; cyclic neutropenia n=1; chemotherapy n=1; juvenile idiopathic arthritis n=1.

Of all the patients, 49.3% were male, with a median age of 13 (interquartile range, 7–17). In 92.3% of the cases, patients were tested due to signs and symptoms of COVID-19. The medical records described close contact with a positive case in 62.5% and a possible close contact in 9%. The most common contacts were family members (62.4%), followed by friends (7.2%) and babysitters (2.4%). The median time from symptom onset to testing was three days (zero to 21 days), with 64.2% of patients getting tested within the first three days of showing symptoms.

The most common symptoms were fever (45.2%), nasal congestion or nasal discharge (44.2%), cough (39.4%), headache (35.3%) and sore throat (31.7%). Other symptoms were present in less than 25% of patients and are described in Table 2. Of all patients, 7.7% were completely asymptomatic, being tested before hospitalization for other causes or due to a contact with a positive person.

**Table 2:** Signs nd symptoms of children and adolescents with COVID-19 in a Brazilian private hospital (n=1303).

Signs and symptoms*	n	%
Fever	589	45.2
Cough	513	39.4
Headache	460	35.3
Coryza	440	33.8
Sore throat	413	31.7
Myalgia	280	21.5
Nasal congestion	248	19.0
Asthenia	243	18.6
Diarrhea	143	11.0
Anosmia	138	10.6
Dysgeusia	105	8.1
Asymptomatic	100	7.7
Dyspnea	63	4.8
Abdominal pain	58	4.5
Nausea	50	3.8
Vomiting	49	3.8
Inappetence	41	3.1
Retro-orbital pain	38	2.9
Chest pain	29	2.2
Otalgia	17	1.3
Skin lesions	15	1.2
Conjunctivitis	11	0.8
Arthralgia	6	0.5
Wheezing	1	0.1

Results are presented in n (%).

* Signs and symptoms were described by patients or caregivers during an Emergency Department visit.

A physical examination revealed that 67.8% of the patients had normal findings. The most frequent abnormality observed was oral mucosa hyperemia, exudate, or swelling on the tonsils, which were present in 27% of the patients. Thirty-one patients (2.3%) had abnormal respiratory auscultation, characterized by wheezing, crackles, and rhonchi. Among them, 13 had signs of respiratory distress. All the findings are described in Table 3.

**Table 3:** Physical examination of children and adolescents with COVID-19 in a Brazilian private hospital (n=1303).

Physical examination*	n	%
Normal	884	67.8
Oral mucosa alterations	353	27.0
Abdominal pain	20	1.5
Snores	17	1.3
Respiratory distress	13	1.0
Altered mental status	10	0.8
Exanthema	10	0.8
Rales	9	0.7
Wheezing	9	0.7
Conjunctival hyperemia	8	0.6
Heart murmurs	2	0.2
Diminished vesicular murmur	1	0.1
Abdominal distention	1	0.1
Peritoneal irritation	1	0.1
Hand/feet edema/exanthema	1	0.1

Results are presented in n (%).

* Physical examination was performed by the attending pediatrician according to regular practice.

A total of 71 patients were tested for coinfections using a multiplex bacterial and viral PCR panel, while 174 patients underwent a rapid test for *Streptococcus pyogenes*, with 17 cases undergoing both tests. Of those tested, 14 patients had viral co-detections, including Influenza B (n=8), rhinovirus (n=2), respiratory syncytial virus (n=1), human coronavirus [HKU1] (n=1), rhinovirus and adenovirus (n=1), influenza B and human coronavirus [NL63] (n=1). Additionally, 14 patients tested positive *Streptococcus pyogenes* using the rapid test. In total, there were 28 cases of identified coinfection, with 20% of patients having viral coinfection and 8% having Streptococcal coinfection among those tested. The patient with both rhinovirus and adenovirus coinfection also tested positive for mycoplasma by RT-PCR. It was found that coinfections were not associated with increased risk of hospital admission (p=0.252). Two patients had positive blood cultures, one for *Burkholderia cepacia* and the other *Staphylococcus epidermidis*, with the latter being considered contamination. The patient with *Burkholderia cepacia* was a newborn with fever without a source, who was submitted to extensive diagnostic workup at admission including blood tests, urine, chest radiography, cerebrospinal fluid, and RT-PCR for SARS-CoV-2. The patient was hospitalized for seven days and initially treated with ceftriaxone, which was later switched to trimethoprim-sulfamethoxazole. She responded well to antibiotics therapy and had no other symptoms of COVID-19 apart from fever. Four patients experienced SARS-CoV-2 reinfection. Among them, there were two males and two females, aged six, 14, 16 and 17 years old. None of them had comorbidities, and none required hospitalization. All patients had total resolution of symptoms between episodes. Only one patient was asymptomatic during the second infection and was tested due to contact with a household member with COVID-19.

Chronic medical conditions were documented in 9.4% of the patient’s medical records. Asthma was the most prevalent comorbidity (6.2), followed by neurologic disorders (1.1%), cardiac diseases (0.9%), genetic disorders (0.5%), immunosuppressive conditions (0.5%), obesity (0.5%), and diabetes (0.2%). The number of patients with obesity is probably underestimated, since we were unable to calculate the body mass index (BMI) due to the absence of height measurement during ED visits. The presence of a comorbidity was associated with a higher rate of readmission to the ED (28.7 *vs.* 14.3%, p<0.001) and hospitalization (8.2 *vs.* 3.0%, p=0.007).

Laboratory tests were performed in 11.7% of the patients, revealing lymphopenia in 40.6%, neutropenia in 16.0%, and thrombocytopenia in 11.3%. Elevated inflammatory markers were also noted, with the C-reactive protein exceeding 50 mg/L in 8.9%, but greater than 100 mg/L in only two individuals (1.4%). D-dimer was elevated in 24.0% of cases, with no cases of thrombosis being diagnosed.

Radiological exams were conducted on 12.1% of the patients. Among the 101 chest radiographs performed, alterations were observed in 29.7% of cases, with perihilar vascular prominence being the most common finding (15.8%). Out of the 43 chest computed tomography (CT) scans performed, alterations were detected in 53.4%, with ground-glass opacities being present in 19 patients (44.1%). Abnormalities were found in 42.8% of the 21 abdominal ultrasounds. All eleven echocardiograms yielded normal results, including one patient diagnosed with myocarditis. Detailed laboratory results and radiological findings can be found in Table 4.

**Table 4:** Laboratory and radiological findings in pediatric patients with COVID-19 in a Brazilian private hospital (n=1303).

Radiological findings			n	%
Chest radiography (n=101)				
	Normal		71	70.2
	Perihilar vascular prominence		16	15.8
	Consolidation		6	5.9
	Hyperinflation		5	4.9
	Interstitial infiltrate		4	3.9
	Pleural effusion		0	0
Chest computed tomography (n=43)				
	Normal		20	46.5
	Ground glass opacities		19	44.1
	Consolidation		4	9.3
	Peribronchial cuffing		3	6.9
	Atelectasis		2	4.6
	Interstitial infiltrate		0	0
	Pulmonary embolism		0	0
Abdominal ultrasound (n=21)				
	Normal		12	57.1
	Adenomegaly		3	14.2
	Appendicitis		2	9.5
	Free fluid		2	9.5
	Other abnormalities		2	9.5
	Echocardiography (n=11)	Normal	11	100.0
Laboratory results (median; IQR)				
	Alanine aminotransferase, U/L (n=92)		19.0 (12.5–27.0)	
	Aspartate aminotransferase, U/L (n=91)		24.0 (18.0–36.0)	
	Blood urea nitrogen, mg/dL (n=104)		22.5 (18.0–28.5)	
	C-reactive protein, mg/L (n=134)		3.7 (1.0–15.7)	
	Creatinine, mg/dL (n=107)		0.7 (0.47–0.86)	
	D-dimer, ng/mL (n=75)		351.0 (235.0–492.0)	
	Eosinophils, x10³/μL (n=150)		42 (9–112)	
	Erythrocyte sedimentation rate, mm (n=7)		16.0 (7.0–18.0)	
	Fibrinogen, mg/dL (n=14)		300.5 (233.0–370.0)	
	Hemoglobin, g/dL (n=150)		13.1 (12.3–14.4)	
	Lactate dehydrogenase, U/L (n=46)		202.5 (170.0–257.0)	
	Lymphocytes, x10³/μL (n=150)		1735 (1022–3005)	
	Neutrophils, x10³/μL (n=150)		3563 (1912– 5865)	
	Platelets, x10³/μL (n=150)		231,000 (190,000–286,000)	
	Total leucocyte count, x10³/μL (n=150)		7015 (5160–9820)	
	Troponin, pg/mL (n=32)		5.0 (5.0–14.5)	

Results are presented in n (%) or median (interquartile range). Normal laboratory values for the tests are alanine aminotransferase ≤19 U/L; aspartate aminotransferase ≤41 U/L; blood urea nitrogen 12–48 mg/dL; C-reactive protein ≤5 mg/L; creatinine <1.2 mg/dL; D-dimer <500 ng/mL; eosinophils 0–650×10³/μL; erythrocyte sedimentation rate 3–13mm; fibrinogen 200–400 mg/dL; hemoglobin 11–14,5 g/dL; lactate dehydrogenase ≤305 U/L; lymphocytes 1500–8500×10³/μL; neutrophils 1500–8500×10³/μL; platelets 150,000–450,000×10³/μL; total leucocyte count 5000–14,500×10³/μL; troponin<5 pg/mL.

After medical evaluation in the ED, 3.5% (n=45) of patients were admitted to the hospital. The median length of stay was three days (interquartile range, 1–32). The reasons for hospital admission were hypoxemia and respiratory distress in nine cases (20.0%), myocarditis investigation in three cases (6.6%), prolonged fever in four cases (8.8%), MIS-C suspicion in two cases (4.4%), and the necessity of intravenous antibiotics for a secondary infection in seven cases (15.5%). Additionally, four patients underwent appendectomy without complications. One adolescent was admitted with renal colic, while another was admitted following an attempted suicide. Both patients had a positive RT-PCR, which was part of an admission protocol during the pandemic, despite not exhibiting COVID-19 symptoms. Two patients required non-invasive ventilation during the ED admission, and no patients required intubation. No deaths were reported, and no MIS-C cases were confirmed in our study population. Further details regarding hospitalization rates according to age group, sex, comorbidities, coinfection, and year of admission are shown in Table 5.

**Table 5:** Hospitalization in children and adolescents with COVID-19 (n=45) in a cohort of 1303 pediatric cases of SARS-CoV-2 infection

Characteristic		Hospitalized patients (n, %)	p-value
Age group (years)			
	0–5 (n=273)	20 (7.3)	<0.001*
	6–11 (n=275)	8 (2.9)	
	12–19 (n=755)	17 (2.3)	
Sex			
	Male (n=642)	23 (3.6)	0.802*
	Female (n=661)	22 (3.3)	
Coinfection†			
	Yes (n=28)	2 (7.1)	0.252‡
	No (n=1275)	43 (3.4)	
Comorbidities§			
	Yes (n=122)	10 (8.2)	0.007‡
	No (n=1181)	35 (3.0)	
Time Period//			
	First Wave – 2020 (n=679)	34 (5.0)	0.001*
	Second Wave – 2021 (n=624)	11 (1.8)	

* Chi-square test;

† Detected coinfections were *Streptococcus pyogenes* (n=14), Influenza B (n=8), rhinovirus (n=2), respiratory syncytial virus (n=1), human coronavirus [HKU1] (n=1), rhinovirus and adenovirus (n=1), influenza B and human coronavirus [NL63] (n=1);

‡ Fisher’s exact test;

§ Comorbidities were asthma, diabetes, obesity, neurologic disorders, cardiac diseases, genetic syndromes and conditions causing immunosuppression;

// The first wave of COVID-19 in Brazil took place during the whole year of 2020 and the second wave began in January 2021.

Readmissions to the ED during the same COVID-19 episode occurred in 15.7% (n=204) of the discharged patients (96.5%). The leading causes were clinical worsening (33.8%), checking extra information about COVID-19 infection and guidance on isolation procedures (17.1%), dyspnea (13.7%), thoracic pain (6.8%), and prolonged fever (3.9%).

There was a higher proportion of asymptomatic patients in the age group of 6–11 years compared to those aged 0–5 years and 12–19 years (13.1 *vs.* 8.8 *vs.* 5.3%, p<0.001). Children aged five years and younger had a higher incidence of fever (64.8 *vs.* 42.2 *vs.* 39.2%, p<0.001), inappetence (9.5 *vs.* 2.2 *vs.* 1.2% p<0.001), and diarrhea (16.5 *vs.* 7.6 *vs.* 10.2%, p=0.002) compared to those aged 6–11 years and 12–19 years. However, they presented a lower prevalence of headache (8.4 *vs.* 33.8 *vs.* 45.6%, p<0.001), myalgia (3.3 *vs.* 14.2 *vs.* 30.7%, p<0.001), sore throat (7.0 *vs.* 27.3 *vs.* 42.3%, p<0.001), cough (32.6 *vs.* 33.8 *vs.* 43.8%, p=0.001), and anosmia (0.7 *vs.* 4.0 *vs.* 16.6%, p<0.001).

Regarding laboratory and radiological findings, children aged 6–11 years had a higher prevalence of abnormal radiographs compared to the 0–5 and 12–19 age groups (46.7 *vs.* 37.5 *vs.* 17.4%, p=0.036). No significant variations between age groups were observed between the results of CT scans and ultrasound scans. The hospitalization rate was higher for children aged 0–5 years (7.3%) compared to the 6–11 (2.9%) and 12–19 (2.3%) age groups (p<0.001).

We also observed that the patients were older during the second wave of COVID-19 (14.0 years *vs.* 12.0 years, p<0.001). During the first wave, patients had higher prevalence of fever (48.2 *vs.* 42.0%, p=0.025), dyspnea (6.6 *vs.* 2.9 %, p=0.002), anosmia (12.2 *vs.* 8.8%, p=0.046), inappetence (4.3 *vs.* 1.9%, p=0.015), and abdominal pain (5.6 *vs.* 3.2%, p=0.037). The only symptom that was more prevalent during the second wave was coryza (37.0 *vs.* 30.8%, p=0.017). No other differences in signs and symptoms were observed. Patients during the second wave had fewer comorbidities (7.5 *vs.* 11.0%, p=0.03) and required less hospitalization (1.8 *vs.* 5.0%, p=0.001). It is worth noting that the COVID-19 vaccine was not yet available for our population during the study period.

## DISCUSSION

This study aimed to investigate the clinical characteristics, outcomes, and factors associated with hospitalization and readmission rates among pediatric patients with SARS-CoV-2 infection. After almost three years of the COVID-19 pandemic, our understanding of the disease in children and adolescents has evolved, as initial beliefs of their lower susceptibility have been disproven. The findings revealed a diverse range of clinical presentations, including a significant proportion (7.7%) of asymptomatic cases, highlighting the importance of implementing widespread testing strategies. Our study further supports the understanding that most infections in children and adolescents are mild to moderate, with a considerable proportion being asymptomatic.^2,12,13^ Comorbidities, such as asthma, were associated with an increased risk of hospitalization and readmission, emphasizing the importance of giving additional attention to pediatric patients with underlying medical conditions. The comparison of different waves of the pandemic revealed variations in patient demographics, comorbidity prevalence, and hospitalization rates, suggesting the evolving dynamics of the virus. Remarkably, we observed higher hospitalization and readmission rates during the first wave compared to the second wave in 2021, even before the initiation of vaccination efforts. Overall, these findings contribute to a better understanding of the clinical profile and outcomes of pediatric patients with SARS-CoV-2 infection.

Notably, our observations align with a Brazilian study that reported no difference in COVID-19 prevalence between children, adolescents, and adults in 133 cities in 2020.^14^ However, our study differed from another conducted in Rio de Janeiro, which reported a much higher prevalence of asymptomatic cases, of 64%, in a population younger than 14 years old.^15^ This discrepancy can be attributed to the fact that asymptomatic patients are less likely to seek medical care in the ED. In 2020, SARS-CoV-2 tests were more readily available in the ED setting compared to routine laboratory testing, potentially leading to a higher proportion of asymptomatic patients seeking evaluation, particularly those with close contact with COVID-19 cases. Interestingly, our findings revealed that the age group of 6–11 years exhibited the highest percentage (13.1%) of asymptomatic patients.

Close contact with a positive case of COVID-19 was documented in 62.5% of our medical records, with the majority being household members, consistent with findings from other case series.^9,15^ It is important to note that the retrospective nature of data collection may have led to an underestimation of this frequency. Additionally, 7.2% of the related contacts were with friends, highlighting that children and adolescents can play a role in the transmission of the infection even when exhibiting mild symptoms.

In an epidemiological study conducted in Brazil, the median time from symptom onset to testing was reported as 10.2 days for all age groups and 8.5 days for the pediatric population.^16^ However, in our study, a higher proportion of patients (64.2%) underwent COVID-19 testing within the first three days of symptom onset. This discrepancy may be attributed to our hospital being a private facility with greater availability of tests throughout the entire pandemic period, allowing for more timely testing compared to the general population.

In other cohorts, the signs and symptoms of COVID-19 were consistent with those observed in our sample. The most prevalent presentations included fever, nasal discharge and/or congestion, cough, headache, and sore throat, while gastrointestinal symptoms were less frequent.^5,9,10,13^ Altered oral mucosa was a notable finding in the physical examination of our sample (27%), which is a common nonspecific manifestation of viral infections in children, as observed in another Brazilian study of children with COVID-19 (20%).^9^ Additionally, we observed a lower incidence of respiratory distress (1.0%) compared to other Brazilian studies that focused on more severe cases, specifically those involving only hospitalized patients.^7,9,10^ Interestingly, we identified four cases of appendicitis in patients with COVID-19 who presented with abdominal pain. It is worth noting that a Brazilian study reported a decrease in appendicitis cases during the pandemic, although there was an increase in complicated cases, likely attributable to delayed medical attention.^17^ In contrast, our cases were not complicated and had timely presentations to the ED, which could potentially be attributed to the higher socioeconomic stratum of the population that sought medical attention despite the ongoing pandemic. However, the incidence of appendicitis in our study could not be evaluated.

Laboratory analyses and imaging studies were performed based on the discretion of the physicians. Most asymptomatic and mild cases did not undergo additional laboratory testing. Given that this is an ED-based study, radiological findings were less common compared to studies focusing on hospitalized children.^9^ The rate of abnormal CT scans in our study was similar to that reported in other studies involving children.^9,13^

A Chinese study reported a 51% co-detection rate in 34 children with SARS-CoV-2, with *Mycoplasma pneumoniae* being the most frequently detected co-pathogen. However, the severity of the co-detection cases was not described.^18^ A study conducted in Singapore in a general hospital found a 1.4% of co-detection in hospitalized patients, all of them adults with mild symptoms and none admitted to the intensive care unit.^19^ We found a 20% rate of viral coinfection, also not related with more readmissions or hospitalization. It is worth mentioning that, in our ED, routine testing for other respiratory viruses using RT-PCR was performed for 5% of all patients with COVID-19, which may have resulted in missed co-detections.

Chronic medical conditions have been associated with an increased risk of severe illness in children with COVID-19.^4,5^ In our study, we observed that patients with comorbidities had a greater risk of ED readmission and hospitalization. However, it is important to note that our overall hospitalization rate was 3.5%, and no deaths occurred, which is consistent with findings from other studies conducted in developed countries.^20^ Conversely, epidemiological studies in Brazil have reported a higher risk of death in children with COVID-19 compared to developed countries.^5,7^ While the number of hospitalizations in our sample is limited to draw definitive conclusions, it is possible that better socioeconomic conditions, appropriate clinical management of chronic diseases, and early visits to the ED during the course of the illness may have contributed to better outcomes in our study population.

Furthermore, two studies compared the prevalence and outcomes of Brazilian children during the first and second waves of COVID-19. The first study, a seroprevalence study in Amazonas, reported a higher positivity rate for COVID-19 antibodies in children during the second wave, although the severity of the disease remained similar in both periods.^11^ The second study reviewed hospital admissions of all children and adolescents in Brazil during the first and second waves. In-hospital mortality rates were comparable between the two waves (7.7 *vs.* 7.6%), but the second wave saw an increase in hypoxemia and intensive care unit admissions among children.^8^ In our study, we observed that patients during the second wave were older (14.0 *vs.* 12.0 years, p<0.001) and presented with milder symptoms such as coryza, while fever and dyspnea were less common. Hospitalizations were also less frequent in the second wave, possibly due to the involvement of patients with fewer comorbidities. Additionally, at the beginning of the pandemic, more children with mild to moderate symptoms might have been hospitalized solely for observation, due to lack of knowledge about the disease and risk of clinical worsening.

Despite the valuable insights gained from our study, several limitations need to be acknowledged. Firstly, the analysis was conducted in a single private hospital with a specific population of high socioeconomic status, limiting the generalizability of the findings to the broader Brazilian population. Moreover, the data were obtained retrospectively from medical records, which may have resulted in missed descriptions of contacts, symptoms, and comorbidities, including obesity. Furthermore, patient selection was based on a positive RT-PCR for SARS-CoV-2, potentially leading to missed cases of MIS-C, a manifestation that can occur up to six weeks after acute infection. Lastly, although we categorized the data based on time to distinguish between waves, we were unable to conduct viral sequencing to confirm the specific virus lineages involved.

In conclusion, this study provides valuable insights into the clinical characteristics and outcomes of pediatric patients with SARS-CoV-2 infection. The findings highlight the diverse range of clinical presentations, including a significant proportion of asymptomatic cases, underscoring the importance of widespread testing strategies. The presence of comorbidities, such as asthma, increases the risk of hospitalization and readmission. Additionally, the study reveals variations in patient demographics, comorbidity prevalence, and hospitalization rates across different waves of the pandemic, indicating the evolving dynamics of the virus. Further research is warranted to assess the impact of immunization and emerging variants on the clinical profile and outcomes of pediatric patients.
